# Quality Standards for the Management of NAFLD: Consensus Recommendations from the British Association for the Study of the Liver (BASL) and British Society of Gastroenterology (BSG) NAFLD Special Interest Group

**DOI:** 10.1016/S2468-1253(22)00061-9

**Published:** 2022-04-28

**Authors:** Stuart McPherson, Matthew J Armstrong, Jeremy F. Cobbold, Lynsey Corless, Quentin M Anstee, Richard J. Aspinall, Stephen T. Barclay, Paul N Brennan, Tessa M Cacciottolo, Robert D Goldin, Kate Hallsworth, Vanessa Hebditch, Kathryn Jack, Helen Jarvis, Jill Johnson, Wenhao Li, Dina Mansour, Mary McCallum, Ashis Mukhopadhya, Richard Parker, Valerie Ross, Ian A Rowe, Ankur Srivastava, Prarthana Thiagarajan, Alexandra I. Thompson, Jeremy Tomlinson, Emmanuel A. Tsochatzis, Andrew Yeoman, William Alazawi

**Affiliations:** 1 Liver Unit, The Newcastle upon Tyne hospitals NHS Foundation Trust and the Translational and Clinical Research Institute, Newcastle University, Newcastle upon Tyne, UK; 2 Liver Unit, Queen Elizabeth University Hospital Birmingham NHS Trust, Birmingham and NIHR Biomedical Research Centre, University of Birmingham, UK; 3 Oxford Liver Unit, Oxford University Hospitals NHS Foundation Trust and NIHR Oxford Biomedical Research Centre, University of Oxford, Oxford, UK; 4 Department of Gastroenterology, Hepatology and Endoscopy, Hull University Teaching Hospitals, Anlaby Road, Hull, UK; 5 Portsmouth Liver Centre, Queen Alexandra Hospital, Portsmouth, UK; 6 Walton Liver Clinic, Glasgow Royal Infirmary, NHS Greater Glasgow and Clyde, Glasgow, UK; 7 Centre for Regenerative Medicine, University of Edinburgh, Edinburgh BioQuarter, Edinburgh, UK; 8 Liver Unit, Cambridge University Hospitals NHS Foundation Trust, Cambridge and Wellcome Trust/MRC Institute of Metabolic Science, Metabolic Research Laboratories, University of Cambridge, Cambridge, UK; 9 Division of Digestive Diseases, Imperial College, London, UK; 10 British Liver Trust, Bournemouth, UK; 11 Nottingham University Hospitals NHS Trust, Nottingham, UK; 12 Population Health Sciences Institute, Newcastle University and The Bellingham Practice, Northumberland, UK; 13 Liver Unit, Queen Elizabeth University Hospital Birmingham NHS Trust, UK; 14 Barts Liver Centre, Queen Mary University London and Barts Health NHS Trust, London, UK; 15 Queen Elizabeth Hospital, Gateshead NHS Foundation Trust, Gateshead and the Translational and Clinical Research Institute, Newcastle University, Newcastle upon Tyne, UK; 16 Digestive Disorders Department, Aberdeen Royal Infirmary, Aberdeen, UK; 17 Leeds Liver Unit, St James’s University Hospital Leeds, Leeds, UK; 18 Leeds Institute for Medical Research, University of Leeds, Leeds, UK; 19 North Bristol Liver Unit, Southmead Hospital, North Bristol Trust, Bristol, UK; 20 Centre for Liver and Digestive Disorders, The Royal Infirmary, Edinburgh, Edinburgh, UK; 21 Oxford Centre for Diabetes, Endocrinology and Metabolism, NIHR Oxford Biomedical Research Centre, University of Oxford, Churchill Hospital, Oxford, UK; 22 UCL Institute for Liver and Digestive Health, Royal Free Hospital and UCL, London, UK; 23 Gwent Liver Unit, The Grange University Health Board, Anuerin Bevan Health Board, Wales, UK

**Keywords:** non-alcoholic fatty liver disease, non-alcoholic steatohepatitis, diabetes, obesity, fatty liver, cirrhosis

## Abstract

Non-alcohol-related fatty liver disease (NAFLD) is common, affecting approximately 25% of the general population. The evidence base for the investigation and management of NAFLD is large and growing, but there is currently little practical guidance to support development of services and delivery of care. To address this, we have produced a series of evidence-based quality standard recommendations for the management of NAFLD, with the aim of driving improvement in patient care. A multidisciplinary group of experts from the British Association for the Study of the Liver (BASL) and British Society of Gastroenterology (BSG) NAFLD Special Interest Group (SIG) produced the recommendations covering: 1. Management of people with, or at risk of, NAFLD before the gastroenterology or liver clinic; 2. Assessment and investigations in secondary care, and 3. Management in secondary care. The quality of evidence for each recommendation was evaluated by the Grading of Recommendation Assessment, Development and Evaluation (GRADE) tool. An anonymous modified Delphi voting process was conducted individually by each member of the group to assess the level of agreement with each statement. Statements were included where agreement was ≥80%. From the final list of statements, a smaller number of auditable key performance indicators (KPIs) were selected to allow services to benchmark their practice. It is hoped that services will review their practice against our recommendations and KPIs and institute service development where needed to improve the care of patients with NAFLD.

## Introduction

Non-alcohol-related fatty liver disease (NAFLD) is common, affecting approximately 25% of the population in many developed countries ^1^. The disease ranges from steatosis to non-alcohol-related steatohepatitis (NASH; fat with hepatocyte injury and hepatic inflammation) and can progress to cirrhosis and liver-related complications including hepatocellular carcinoma (HCC) and liver failure ^2^. Individuals with NAFLD have an increased risk of overall mortality compared with the general population, and common causes of death include cardiovascular disease, malignancy and liver-related complications ^3–5^. The majority of the estimated 14.1 million individuals with NAFLD in the UK remain undiagnosed and worryingly the prevalence of advanced fibrosis/cirrhosis is projected to double to 1 million individuals by 2030 ^6^. Despite the high prevalence of NAFLD in the population, recognition and management of the condition is variable across the UK. One multicentre study from England found a large variability in the primary and secondary care management of NAFLD, with clear deficiencies identified in primary care investigations, fibrosis staging, provision of lifestyle treatments, and assessment and management of cardiovascular risk factors ^7^. There is therefore a clear need to improve the holistic management of patients with NAFLD in order to achieve better outcomes.

## Aim

The purpose of this work was to develop a series of quality standard recommendations from a multidisciplinary panel of experts for the management of patients with NAFLD to provide a standardised management approach, with the ultimate objective of reducing variability in care nationally. In addition, we have developed a series of auditable key performance indicators (KPIs) to measure practice against to help drive service improvement.

## Methods

A group of experts from the British Association for the Study of the Liver (BASL) and British Society of Gastroenterology (BSG) NAFLD Special Interest Group (SIG) developed the recommendations. SM and WA chaired the group. All members of the NAFLD SIG were invited to participate via email and those expressing an interest were included in the working group. Ultimately, the working group included a multidisciplinary team of 29 individuals from hepatology, diabetes, dietetics, hepatology specialist nursing, pathology, primary care, psychology, pharmacy and physiotherapy. The group also included a representative from the British Liver Trust (VH). The group was sub-divided into three working groups that led the writing of draft recommendations for one of three parts of the document: 1. Management of people with, or at risk of, NAFLD before the gastroenterology or liver clinic (lead LC); 2. Assessment and investigations in secondary care (lead MJA), and; 3. Management in secondary care (lead JFC).

Each group produced a list of important topics in the NAFLD diagnosis and management pathway to address within the standards document. A literature search was conducted using PubMed Medline to identify relevant original research papers and existing guidelines to end of June 2021. The specific statements were then made by each group, informed by the quality of the evidence evaluated in line with the Grading of Recommendation Assessment, Development and Evaluation (GRADE) tool. SM and WA amalgamated the draft statements from the three working parties and removed any duplication. An anonymous modified Delphi voting process was conducted individually by each member of the working group using an online survey tool to assess the level of agreement with each statement on a five-point scale (strongly disagree [SD], disagree [D], neutral [N], agree [A] or strongly agree [SA]). Given the working group was multidisciplinary, members could abstain from questions that related to areas out with their usual clinical practice (for example, a dietician may not feel qualified to make clinical decisions regarding when to perform a liver biopsy). After each round of voting, statements were redrafted where necessary through a combination of discussions via teleconference meetings and email. ‘Agreement’ was defined when statements received a score of ‘strongly agree’ or ‘agree’. Statements were included where agreement was ≥80%, after exclusion of any abstains. The result of this process produced a series of recommendations, with a corresponding level of expert agreement and grading of the relevant evidence.

From this final list of statements, a smaller number of auditable KPIs were selected to allow services to benchmark their practice. The KPIs were chosen based on their potential to influence patient outcomes as well as being easily measurable.

## Quality standards

Following the Delphi voting process and the review of evidence, 34 quality standard recommendations (Table 1) were made covering the management of NAFLD in the community and in secondary care. A review of the supporting evidence is shown below. In addition, 11 auditable KPIs have been developed and are shown in Table 2.

### Management of people with, or at risk of, NAFLD before the gastroenterology or liver clinic

#### Identification of people with NAFLD

Use of defined pathways for the investigation of suspected liver disease has been shown to increase the diagnosis of significant liver disease and reduce unnecessary referrals ^8,9^. Therefore, services should have an agreed local clinical pathway for the investigation of suspected liver disease that includes an assessment for liver fibrosis using available non-invasive liver fibrosis tests (Recommendation 1, Table 1). Key aspects to consider when developing pathways are described below.

NAFLD is considered the hepatic manifestation of the metabolic syndrome (defined as any three of the following: impaired fasting glucose or T2DM; hypertriglyceridemia; low HDL; increased waist circumference or high blood pressure) ^10^. As well as NAFLD being highly prevalent in those with T2DM or the metabolic syndrome, the presence of these risk factors is associated with more progressive liver disease in NAFLD ^11^. Therefore, consider the possibility of liver fibrosis due to NAFLD in those with Type 2 diabetes (T2DM) or the metabolic syndrome (Recommendation 2, Table 1).

Proactive assessment for the presence of liver fibrosis in patients at risk can permit earlier identification of significant liver disease ^12–14^. The ‘Scarred Liver Project’ ^15^, which offered community fibrosis testing to people with diabetes or obesity alongside those with hazardous alcohol consumption, identified 3688 patients at risk from a cohort of 25,018. Overall, 20% of at-risk individuals who attended a follow-up clinic had evidence of significant liver disease. Furthermore, a study including FIB-4 (discussed below) testing in annual diabetic reviews in primary care followed by transient elastography in those with an indeterminate or high FIB-4 found that 4.5% of the cohort had previously undiagnosed advanced liver disease, defined as imaging, endoscopic or biopsy evidence of cirrhosis, portal hypertension or HCC ^16^.

Evidence to support a case finding strategy amongst people with significant risk factors is currently limited and remains an area of divergence between current European and North American clinical guidelines ^17,18^. However, it is likely that a fibrosis risk-based approach in primary care may be more successful - and cost-effective - for the early identification of liver disease, than reliance on abnormal liver function or incidental finding of steatosis on imaging ^19^. However, it is also important to acknowledge that greater efforts to investigate and identify advanced liver disease in this group may result in a significant increase in primary and secondary care workload, and local service development considerations should be planned accordingly.

#### Liver blood tests

Liver blood tests, including alanine aminotransferase (ALT), alkaline phosphatase (ALP), bilirubin and gamma glutamyl transferase (GGT), are frequently tested as part of routine clinical investigation in primary care. Whilst unexplained persistently abnormal liver blood tests should always be investigated, normal liver blood tests do not exclude NAFLD or significant fibrosis ^17,18^. In a study of 223 patients with biopsy-proven NAFLD, ALT more than two times the upper limit of normal (> 70 IU/L) had a sensitivity of just 50% and specificity of 61% for NASH, and sensitivity 40% and specificity 58% for advanced fibrosis ^20^. Moreover, the serum ALT level typically falls as liver fibrosis progresses and patients with cirrhosis frequently have a normal range ALT level ^21^. Therefore, do not rely on abnormal liver blood tests to prompt consideration of liver disease. However, persistently unexplained abnormal liver blood tests should always be investigated. (Recommendation 3, Table 1)

Patients with abnormal liver blood tests should be evaluated in line with national recommendations ^22^, with a focussed history eliciting risk factors for chronic liver disease including unhealthy alcohol consumption, the presence of metabolic risk factors and a detailed drug history. Automated systems, such as intelligent liver function tests (iLFT), can streamline the investigation of abnormal liver blood tests giving a full panel of results, suggested diagnosis and advice for further management ^8^. This approach increases the diagnosis of liver disease and is highly cost effective.

#### Liver fibrosis assessment

An increasing body of evidence has demonstrated advancing liver fibrosis as the key predictor of liver-related events and mortality in patients with NAFLD ^4^. Patients with advanced fibrosis and cirrhosis (equivalent to Brunt fibrosis stage 3 or 4) are at increased risk of complications of chronic liver disease, decompensation, liver transplantation and death in the short- to medium-term ^5,23–25^.

Since liver blood tests and ultrasound poorly discriminate fibrosis stage, primary care pathways for patients with NAFLD should assess for the presence of advanced fibrosis and cirrhosis using validated non-invasive fibrosis tests ^22^. Accordingly, we suggest the finding of liver steatosis on ultrasound, or unexplained abnormal liver blood tests, should prompt risk assessment for liver fibrosis (Recommendation 4, Table 1). Use validated widely available non-invasive tests with high negative predictive value to risk assess for significant liver fibrosis in the community (Recommendation 5, Table 1). Refer patients stratified as high risk for advanced fibrosis or cirrhosis to a hepatologist. For patients stratified as indeterminate risk, offer further discriminatory tests (such as transient elastography or ELF test) or refer for further evaluation (Recommendation 6, Table 1).

Indirect biomarkers of liver fibrosis such as FIB-4 and NAFLD Fibrosis score have been shown to have prognostic value for long term outcomes and are validated against liver biopsy with good negative predictive value to rule-out advanced liver fibrosis in NAFLD ^21,26,27^. They combine routinely applied laboratory tests with clinical parameters and are easily applicable. Patients with a FIB-4 index < 1.3 (or < 2.0 if over 65 years) or NFS <-1.455 (or < 0.12 if over 65 years) have a low probability of having advanced fibrosis and can be reassured ^28–30^. Of note, neither the FIB-4 nor the NFS have been validated in patients under 35 years of age ^29^. Evidence for the performance of other biomarkers such as Enhanced Liver Fibrosis test (ELF) or transient elastography (discussed below) in young patient cohorts is also lacking so non-invasive tests should be interpreted with caution in this age group.

Exemplar pathways exist as recently reviewed ^31^. The Camden and Islington NAFLD pathway stratified fibrosis in over 1450 patients with NAFLD over two years using FIB-4 in all patients and ELF test in FIB-4 indeterminate cases ^9^. Patients identified as high-risk for advanced fibrosis and cirrhosis were re-assessed in secondary care and resulted in a 5-fold increase in detection of advanced fibrosis, 3-fold increase in the detection of cirrhosis and 81% reduction in referrals of patients with mild disease in comparison to the absence of any defined care pathway.

A number of cost-effectiveness studies have demonstrated the benefit of such risk stratification strategies. The use of serum markers and elastography, either alone or in combination has been shown to be clinically effective and cost saving compared to standard care ^32–34^.

Patients stratified as high risk for advanced fibrosis or cirrhosis whose liver disease is not outweighed by comorbidities or performance status should be referred to a hepatologist or gastroenterologist with an interest in liver disease for further evaluation of their condition.

For patients stratified as indeterminate risk for advanced fibrosis or cirrhosis using simple non-invasive tests (NIT), the result is neither sensitive nor specific enough to confidently rule in or rule out advanced fibrosis or cirrhosis. A second tier liver fibrosis test such as a direct collagen biomarker (e.g., ELF) or elastography (e.g., transient elastography) should be offered ^22^. Although the optimum pathway has not yet been determined, liver stiffness measurement (LSM) by transient elastography > 8 kPa or an ELF > 9.5 has been used to prompt referral to secondary care liver services among existing pathways ^32^

Ideally, second-stage non-invasive testing should be delivered in the community to reduce the unnecessary referral to secondary care for some individuals with a false positive simple NIT. However, where these services are not available in the community, patients should be referred to secondary services for further workup ^32^.

**Figure 1:**
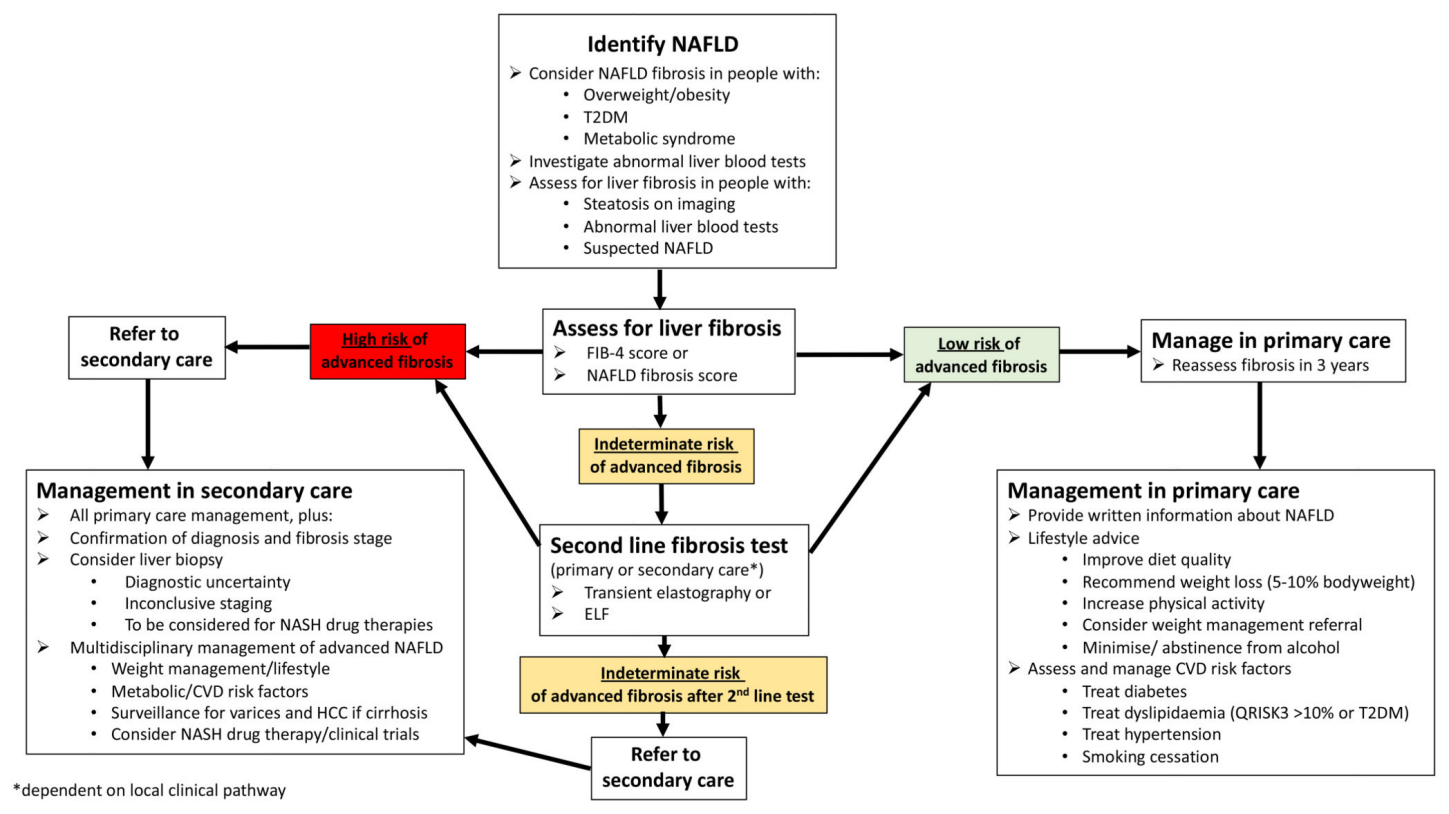
An overview of the clinical management of individuals with NAFLD in primary and secondary care. ELF=Enhanced Liver Fibrosis test. NAFLD=non-alcoholic fatty liver disease. NASH=non-alcoholic steatohepatitis.

The overwhelming majority of liver-related complications in patients with NAFLD occur in those with cirrhosis ^4^. NITs have high negative predictive values for advanced fibrosis so can reliably exclude the presence of cirrhosis. Moreover, long-term follow-up studies have shown that patients with low-risk NITs have a very low risk of liver-related events in the short- to medium-term, and their main morbidity is cardiovascular disease and non-hepatic malignancy ^26,35^. One recent long term follow up study of 1057 patients showed that individuals with NAFLD and a FIB-4 score <1.3 had a very low incidence rate for liver-related events of 2.6 per 1000 patient years ^36^. Therefore, for these patients the focus should be on lifestyle advice and cardiovascular risk reduction with the aim of improving their overall quality and length of life (Figure 1 and NAFLD management section below). NAFLD can progress to advanced fibrosis in a significant proportion of patients in the medium-term, particularly those with, or who develop, T2DM and those who gain weight ^37^. Therefore, individuals with these risk factors should be targeted for more proactive lifestyle modification, optimisation of treatment for T2DM and cardiovascular risk reduction. Accordingly, manage those at low risk of significant fibrosis in the community, with focus on lifestyle advice and cardiovascular risk reduction. Reassess using non-invasive tests after 3 years. (Recommendation 7, Table 1)

Given fibrosis progresses in a significant proportion of individuals with NAFLD, repeated fibrosis assessment is required in individuals who remain at risk. The interval for a repeat fibrosis assessment is proposed to be between 1-3 years in the recent EASL CPG on non-invasive fibrosis assessment ^38^. Taking into account that the time to progression by one stage of liver fibrosis is estimated between 7 and 14 years ^39^ a 3-year interval is a realistic timeframe to reassess fibrosis. In individuals with NAFLD and no evidence of significant liver fibrosis who have no risk factors for fibrosis progression and achieve weight loss goals it may be appropriate to extend this interval for fibrosis reassessment to 5 years. An automatic recall will need to be built in the patients’ electronic records ^32,34^.

#### Service development

Secondary care liver services and community services should collaborate on audit, research and education to share knowledge, strengthen links and encourage service and quality improvement and involve patients as part of this as appropriate (Recommendation 8, Table 1). Healthcare partnerships between primary and secondary care can be helpful to strengthen collaboration and improve outcomes for patients with chronic liver disease. A multi-disciplinary liver working group comprising of local hepatology/gastroenterology leads, primary care leads for liver disease, strategy leads for commissioners, public health doctors and public and patient involvement (PPI) representatives can lead on local service improvement initiatives for patients with chronic liver disease. This approach enables development of strategies tailored to local resources and allows collaboration for development of education programmes for healthcare professionals, audit of interventions and research.

### Assessment and Investigations in Secondary care

#### Assessment for additional causes for steatosis

Patients with NAFLD should be assessed for additional causes of steatosis (e.g. drugs and alcohol) and undergo investigations for other causes of liver disease (i.e. completion of blood aetiology screen) if not already undertaken in primary care (Recommendation 9, Table 1). Consumption of alcohol at unhealthy levels is associated with high rates of hepatic steatosis, with half of heavy drinkers of normal BMI and over 90% of obese heavy drinkers affected ^40^. In addition, alcohol use amongst non-heavy drinkers is also associated with an increased risk of steatosis, particularly amongst those who binge drink ^41^. An accurate alcohol history is therefore required, both to identify unsuspected alcohol-related liver disease, and to facilitate tailored alcohol advice to those who drink within recommended limits. Screening for alcohol misuse, including identification of binge drinking, may be standardised by incorporating a tool such as the AUDIT-C questionnaire ^42^ into assessment of patients with suspected NAFLD. Therefore, patients with NAFLD should have a detailed alcohol (such as AUDIT-C), illicit drug and smoking history documented (Recommendation 10, Table 1)

Less commonly other drugs may be the precipitant for hepatic steatosis, with around 2% of cases of NAFLD attributable to prescribed medication ^43^. Drugs implicated in steatosis span many classes including antiarrhythmics (amiodarone), anticonvulsants (carbamazepine, sodium valporate), analgesics (NSAIDs), glucocorticoids, anti-metabolites (5-Fluorouracil, Methotrexate), oestrogen receptor modulators (tamoxifen) and antiretrovirals (efavirenz) ^44,45^. Initial assessment of patients with suspected NAFLD should therefore include a drug history, with consideration given to whether medication may be either the precipitant or a cofactor for steatosis. In addition, documenting a smoking history is important, especially given that cigarette smoking is associated with progressive fibrosis and cardiovascular disease ^46,47^

A comprehensive metabolic and serological screen should be undertaken (autoimmune, viral, iron/copper studies, alpha-1 antitrypsin) to consolidate the diagnosis of NAFLD and exclude co-existent liver disease ^22^. Genotype 3 strains of viral hepatitis C are associated with increased rates of steatosis ^48^, reinforcing the need for viral serology as part of a liver screen for all patients undergoing evaluation for suspected NAFLD.

#### Treatment history and medicines use review

Individuals with NAFLD frequently have co-morbidities and as a result polypharmacy is common ^49^. Therefore, a review of prescribed medications, use of over-the-counter medications and alternative/complementary medicines should be undertaken. As discussed above, commonly prescribed agents used to treat other conditions may contribute to hepatic fat accumulation (e.g. amiodarone, tamoxifen) or accelerate progression (e.g. methotrexate). Whilst the published literature is at times conflicting, capturing use of methotrexate (including duration of exposure and cumulative dose) is particularly relevant as it is a potential cofactor promoting presence of a persistent transaminitis and/or an increased risk of advanced fibrosis/cirrhosis in patients who are overweight or diabetic ^50,51^. Consider recommending the discontinuation of relevant hepatotoxic medications after risk assessment, involving other relevant specialists as necessary. Moreover, it is important to conduct a medicines use review because discrepancies between patient-reported and medical record documented medications exist in more than 50% of patients with liver disease, particularly those taking more than five medications ^52^. Accordingly, we suggest practitioners should document a treatment history and medicines use review. The rationalisation of medicines that may accelerate disease progression should be considered. (Recommendation 11, Table 1).

#### Assessment of dietary habits and physical activity

Poor diet and limited physical activity are common in people diagnosed with NAFLD ^53–55^. Nutrition surveys are difficult to interpret, but poor dietary choices are associated with an increased risk of NAFLD, for example consumption of fructose-rich soft drinks ^53,56,57^ and animal protein ^58–60^. Understanding of patients’ diet can allow for dietary advice to improve health. Changes to diet including calorie restriction, carbohydrate restriction or fat reduction can improve NAFLD ^61,62^ and encouraging a Mediterranean diet may be more acceptable to patients ^63,64^. Increasing physical activity can improve NAFLD ^61,65,66^, this may be aerobic exercise and/or resistance training in those with limited mobility ^67^. Therefore, an assessment of dietary habits and physical activity levels should be obtained (Recommendation 12, Table 1)

#### Non-invasive liver fibrosis assessment

Patients with NAFLD should undergo sequential use of a simple non-invasive test (e.g. FIB-4) and specialist non-invasive tests (e.g. ELF, transient elastography, or ARFI) to assess the severity of fibrosis. (Recommendation 13, Table 1). Ideally, an initial fibrosis assessment should have been undertaken in primary care and patients with suspected advanced fibrosis referred into secondary care as they have the greatest risk of hepatic morbidity ^4^. In secondary care, fibrosis stage should be confirmed, or second line testing conducted with more specialist tests in individuals where simple non-invasive tests are indeterminate. The performance of biomarkers may be influenced by the prevalence of the target condition in the population being assessed so clinicians should consider the likely disease prevalence in their practice setting and adopt suitable test thresholds to achieve the desired performance ^68^. In general, use of simple non-invasive tests excludes the majority of cases with mild fibrosis ^9,21,29^. Addition of a second line test (e.g. ELF, transient elastography or ARFI), further reduces the number of cases with an indeterminate score, allowing liver biopsy to be reserved for use in a minority of patients where it adds additional useful information ^26,69,70^. Such two-stage care pathways are currently considered to provide the most robust means for the risk-stratification of patients with NAFLD ^31,71^. Ongoing regular non-invasive fibrosis reassessment should be considered every 1-3 years to monitor response to treatment or for fibrosis progression ^38^.

#### Liver biopsy

Given the high prevalence of NAFLD in the general population, fatty liver frequently co-exists with other liver diseases (especially viral hepatitis, autoimmune and haemochromatosis), thereby necessitating a biopsy to understand their relative contributions to the patient’s condition ^72^. Furthermore, the diseases may be synergistic e.g., iron overload and NAFLD ^73^.

Liver biopsy remains the standard for diagnosing NASH and assessing disease activity (inflammation; ballooning) as there are no approved non-invasive radiological or serological markers specific for NASH. International drug authorities (FDA and EMA) therefore continue to recommend that phase 2/3 clinical trials utilise liver biopsy to confirm the diagnosis and assess the grade and stage of the disease, to determine trial entry, as well as act as a primary trial endpoint. There has been a recent debate about the role and reliability of histology in this setting ^74,75^.

In the event that there is discordance between non-invasive fibrosis markers, a biopsy may be required to stage fibrosis; and most importantly rule in or out cirrhosis.

Patients with NAFLD should therefore be considered for a liver biopsy: A. if there is diagnostic uncertainty (other aetiologies/overlap conditions); B. to evaluate the severity of NASH and be considered for potential drug therapies (including clinical trials); C. to determine the stage of liver fibrosis where non-invasive tests are inconclusive to aid with future management (e.g., F4 for HCC surveillance) (Recommendation 14, Table 1).

The Royal College of Pathologists has developed guidelines for the processing, staining and reporting of liver biopsies ^76^ and these should be followed. The pathologists who report these biopsies should be active participants in the EQA scheme run by the UK Liver Pathology Group. It is recommended that the biopsy reporting should include the individual components of either the NASH Clinical Research Network criteria (NAS) or steatosis, activity fibrosis (SAF) score with the choice of score being made by agreement with the clinicians ^77,78^. Accordingly, liver biopsies should be processed, stained and examined according to the RCPath guidelines and reported by pathologists who participate in the liver EQA scheme using a validated score such as the NASH CRN or SAF score. (Recommendation 15, Table 1)

#### Surveillance for liver-related complications

Individuals with NAFLD cirrhosis are at risk of complications of cirrhosis similar to other liver diseases. Therefore, patients should be screened for gastro-oesophageal varices and HCC, in accordance with national/international recommendations. ^79–81^. The Baveno VI exclusion criteria to guide screening for varices (LSM <20kPa and platelet count >150 x10^9^/L) or the expanded Baveno VI criteria (LSM <25kPa and platelet count >110 x10^9^/L) have been validated in NAFLD ^82–84^, allowing endoscopy to be safely avoided in these selected patients. At present there is no prospective evidence to support screening for HCC in patients with NAFLD without cirrhosis. Accordingly, we suggest patients with NAFLD cirrhosis should be offered surveillance for complications of cirrhosis, including HCC and varices, in accordance with national/international recommendations. The Baveno VI exclusion criteria should be considered as a non-invasive tool to rule out the presence of varices requiring treatment. (Recommendation 16, Table 1)

While a higher prevalence of advanced colorectal neoplasms has been reported in patients with NAFLD ^85^, there is currently no evidence to recommend surveillance in these individuals over and above the National Health Service bowel cancer screening programme.

#### Cardio-metabolic risk assessment

Patients with NAFLD have increased cardiovascular-related morbidity and mortality, largely as a result of the association between NAFLD and the metabolic syndrome ^5,86^. Cardiovascular disease (CVD) is the leading cause of death among patients with NAFLD, accounting for over a third of the deaths ^5^. The risk of CVD mortality increases with disease severity, with higher rates in those with biopsy-confirmed NASH (2-fold) and in particular advanced fibrosis ^87^. Even though debate remains over the true causal relationship between NAFLD and CVD ^86,88^, the overall consensus is that traditional cardiovascular risk factors should be actively assessed and modified (*where possible*) in order to improve clinical outcomes in patients with NAFLD. Modifiable risk factors of CVD include smoking, hypertension, high non-high-density lipoprotein (HDL) cholesterol, lack of physical activity, unhealthy diet, alcohol intake above recommended levels and overweight/obesity. In addition, there should be an increased awareness of non-modifiable risk factors including older age, male gender, family history of CVD, and ethnic background (esp. South Asian origin) when evaluating CVD risk in patients with NAFLD ^89^.

Several risk scores (e.g., Framingham, QRISK) have been suggested over the years to estimate risk of CVD at 10 years in the general population. UK guidelines ^89^ currently recommend the QRISK-3 assessment tool (which includes ethnicity) in all individuals over 40 years of age who have no prior history of CVD. Although these guidelines are for asymptomatic individuals and are yet to be validated in patients with NAFLD, there is no reason to suggest that QRISK-3 would not be applicable in NAFLD clinics to guide primary prevention with pharmacological therapy (e.g., lipid lowering therapy) ^89,90^. Therefore, people with NAFLD should undergo systematic assessment of cardiovascular risk factors including use of an objective risk score (e.g., QRISK-3) (Recommendation 17, Table 1). Individuals with 10% or greater 10-year risk of developing CVD should be offered HMG-CoA reductase inhibitor (“statin”) therapy for primary prevention in line with existing guidelines for people at risk of CVD ^89 91^.

NAFLD has a strong relationship with multi-organ insulin resistance, most notably the liver, muscle and adipose tissue. NAFLD is associated with a 2-5 fold increased risk of developing T2DM after adjustment of several metabolic and lifestyle confounders ^92^. We therefore suggest that patients with NAFLD should be screened annually for Type 2 Diabetes (using HbA1c), hypertension and dyslipidaemia (Recommendation 18, Table 1). The American Diabetes Association recommends annual screening for T2DM in individuals considered to be at high risk of T2DM (i.e. those with obesity, older age, 1^st^ degree relative with diabetes) ^93^. NICE guidelines recommend annual screening for T2DM amongst individuals with risk factors *and* evidence of an elevated plasma fasting glucose *and* pre-diabetes (defined as an HbA1c 42-47 mmol/mol (6.0-6.4%))^94^. For those with an HbA1c of less than 42 mmol/mol they recommend a reassessment in 3 years ^94^. Given NAFLD is recognised as a high-risk group for T2DM ^34^ and for simplicity we advocate annual screening for diabetes in patients with NAFLD. A HbA1c of 48 mmol/mol (6.5%) or above is diagnostic for T2DM ^95^. The utility and convenience of blood testing for HbA1c in the out-patient setting, favours its use over that of fasting glucose sampling and/or 75g oral glucose tolerance test

Patients with NAFLD are at risk of proatherogenic dyslipidaemia characterized by high triglycerides, increased very-low- density lipoprotein (LDL), and a higher concentration of small dense LDL coupled with low high-density lipoprotein (HDL) concentrations ^96^. In addition, many prospective studies have shown that NAFLD is an independent risk factor for systemic hypertension (3-fold increase versus non-NAFLD) ^97^, which itself when left uncontrolled (clinic blood pressure >130/85) is a major risk of all-cause and CVD-related mortality ^98^. Not only are these metabolic conditions treatable (e.g., statin, antihypertensives), but they can be used as clinical markers to predict those patients with NAFLD who are at risk of underlying NASH and progressive fibrosis ^99^.

### Management of NAFLD in secondary care

#### Lifestyle management

##### Smoking

People with NAFLD should be asked about smoking and, if they smoke, should be advised to stop and offered referral to smoking cessation services. (Recommendation 19, Table 1). CVD is the most common cause of death among patients with NAFLD ^5^. Tobacco smoking markedly increases risk of cardiovascular, neoplastic and respiratory diseases, leading to increased all-cause morbidity and mortality. Smoking cessation dramatically reduces age-specific mortality rates ^100^. People with NAFLD should be asked about their current and past cigarette smoking history. People who currently smoke should be advised to stop and offered assistance in stopping and referred to smoking cessation services ^101^.

##### Physical activity

People with NAFLD should be advised on benefits of regular exercise; a baseline assessment of physical activity should be made and individualised advice given to increase physical activity (Recommendation 20, Table 1). Both aerobic and resistance training are effective in reducing liver fat, independent of weight loss ^67^. The two types of exercise have different characteristics that make them suitable for different patients: resistance exercise has a lower cardiorespiratory demand so may be preferential for patients with poor baseline fitness or those with comorbidities that prevent participation in aerobic exercise. Recommendations for exercise in NAFLD include 150-300 min/week of moderate intensity aerobic exercise performed over a minimum 3 days/week and resistance exercise on at least 2/days ^102^. Most importantly, advice should be individualised to promote adoption and long-term adherence to the physical activity/exercise intervention, which may be facilitated by behaviour change strategies ^103^.

##### Alcohol consumption

The widely accepted European definition of NAFLD emphasises the absence of excessive alcohol consumption (stated as ≥30 g per day for men and ≥20 g per day for women) and a quantitative alcohol history is essential for diagnosis ^17^. Patients with NAFLD should have a regular reassessment of their alcohol consumption (Recommendation 21, Table 1). There are additive and synergistic interactions between alcohol and cardiometabolic risk factors in the progression of fatty liver disease ^104^. Much of the evidence linking alcohol to health outcomes relies on cohort studies where alcohol consumption was measured only once at baseline but it is recognised that alcohol consumption fluctuates widely over the life course ^105^. In view of this, we would recommend that patients with NAFLD should have a quantitative alcohol history taken at regular intervals.

The transition from compensated to decompensated disease is associated with greatly increased morbidity and mortality. In people with alcohol-related liver disease, continued drinking is a stronger risk factor for decompensation than any histological or laboratory parameters ^106^. As cofactors, both alcohol use and obesity have been shown to correlate with progression of portal hypertension in chronic liver disease ^107,108^.

In patients with NAFLD who do not have cirrhosis, the 2016 NICE clinical guideline reported insufficient evidence to restrict alcohol consumption beyond the national recommended advisory limits ^34^. However, alcohol can be a source of additional dietary calories ^109^ and minimising use may avoid further weight gain with worsening of metabolic risk factors. Accordingly, abstinence from alcohol should be strongly recommended to patients with NAFLD and cirrhosis. Patients with pre-cirrhotic NAFLD should be advised that alcohol consumption may accelerate disease progression and so should minimise or abstain from alcohol to reduce the risk of disease progression. (Recommendation 22, Table 1).

##### Dietary advice and weight management

Tailored dietary advice should be given with the aim of 5-10% body weight loss through a calorie deficit including, but not limited to, reduction of refined carbohydrates and processed foods, and increased consumption of vegetables, lean protein sources and fish. Referral to weight management services should be considered, especially when weight loss goals have not been achieved. (Recommendation 23, Table 1). Weight reduction, through caloric restriction, is fundamental to improving disease severity. Whilst 5% body weight loss improves steatosis ^110^, 7-10% is required to positively affect NAFLD activity score and fibrosis ^111,112^. Histological changes achieved by weight loss show a dose-response relationship, with >10% body weight reduction associated with NASH resolution and improvement of fibrosis by one stage ^113^.

Dietary composition can affect hepatic fat accumulation, particularly if high in saturated fats, processed foods and refined sugars. However, histological improvement is dependent on the degree of weight loss rather than the method used to achieve it. To date, studies assessing specific diets in NAFLD have been small and limited in their outcomes assessment so the optimal diet for NAFLD is not known. Given its documented potential to reduce hepatic fat content and have positive effects on cardiovascular risk, the Mediterranean diet is the most widely recommended diet for NAFLD ^114^. Taking an individualised approach to promote weight loss and improve diet quality is likely to be most effective approach. Therefore, referral to weight management services should be considered for specialist dietetic support, pharmacological or surgical intervention, particularly when dietary goals have not been achieved.

Referral for consideration of bariatric surgery should be considered in NAFLD patients with obesity and who meet the eligibility criteria for bariatric surgery according to national recommendations (Recommendation 24, Table 1). NAFLD, across the entire spectrum of severity (including cirrhosis) is highly prevalent in patients with severe obesity ^115^. In addition to the established benefits of bariatric surgery (sleeve gastrectomy or roux-en-Y gastric bypass) on morbidity and mortality, there is robust histological evidence to demonstrate improvements in NAFLD liver histology ^116^. Bariatric surgery in patients with NASH can result in NASH resolution; a prospective study of 109 individuals with NASH found 85% (95% CI, 75.8-92.2) had resolution on biopsy a year post-bariatric surgery ^117^. A more recent meta-analysis found obesity surgery improves steatosis and steatohepatitis in 88% (95% CI, 80-94) and 59% (95% CI, 38-75) of patients respectively, and fibrosis in 30% (95% CI 21-41) ^118^. Furthermore, bariatric surgery also improves overall mortality from cardiovascular and malignant causes.

The decision to undertake bariatric surgery needs to be carefully considered in an appropriate multidisciplinary setting. Patients with cirrhosis can undergo bariatric surgery safely ^119^ and in small case series, bariatric surgery combined with liver transplantation has been performed ^120^_._

#### Management of chronic liver disease

##### Secondary care follow-up for patients with NAFLD

Patients with NAFLD who are at risk for development of liver-related complications include those with cirrhosis and significant-advanced fibrosis. These patients need to be managed in a secondary care setting, akin to those with other aetiologies. Regular surveillance of patients with cirrhosis with 6-monthly ultrasound is one of the quality standards laid down by NICE ^79^ and allows early diagnosis of hepatocellular carcinoma and prompt treatment, which can improve the individual’s survival ^121^. Regular monitoring also allows early detection and treatment of liver-related complications such as ascites, varices and hepatic encephalopathy and appropriate referral for liver transplantation once clinical thresholds are met ^122^. The process should be individualized wherein those with significant co-morbidities and poor performance status are counselled against active monitoring ^123^.

Individuals with NASH who have significant (F2), advanced fibrosis (F3) or cirrhosis (F4) are at risk of progression to end stage liver disease and potential complications in the medium to long-term ^4,5,37^. Therefore, secondary care follow up may be appropriate for these individuals to consider specific treatment for NASH or investigational drugs ^17,34^. Accordingly, we suggest that people with NAFLD who are at significantly increased risk of disease progression and potential risk of liver related complications should continue to be managed in the secondary care setting. Such patients include those with cirrhosis or significant-advanced fibrosis whose liver disease is not outweighed by comorbidities or performance status. (Recommendation 25, Table 1)

##### Liver transplantation

Patients with decompensated liver disease caused by NAFLD should be considered for transplant assessment (Recommendation 26, Table 1). This includes patients with jaundice, ascites, hepatic encephalopathy, variceal bleeding, or hepatocellular carcinoma within the accepted UK liver transplant criteria ^124^. Factors that may influence the decision to refer for assessment include the presence of life-limiting comorbidities or recent (within 5 years) extrahepatic malignancy (except skin) that would be contraindications to transplantation.

#### Management of cardio-metabolic risk factors

##### Hypertension

NAFLD is strongly associated with an increased risk of hypertension ^97^. Approximately 50% of patients with hypertension have NAFLD and correspondingly the prevalence of hypertension is significantly higher in patients with NAFLD, independently of other cardio-metabolic risk factors ^125^. In addition to lifestyle advice, pharmacological therapy should be offered to all patients, with the aim of optimising blood pressure and thereby reducing cardiovascular risk ^126^. We recommend that patients with hypertension should be managed in accordance with NICE guidelines (Recommendation 27, Table 1).

##### Dyslipidaemia

Despite the risk of progressive liver disease, the leading cause of death in patients with NAFLD is CVD ^127^. This increase in mortality from CVD in patients with NAFLD is related to shared cardiometabolic risk factors. Patients with more advanced degrees of fibrosis and T2DM demonstrate a higher propensity to CVD ^128,129^. Patients with NAFLD (with or without T2DM) should have cardiovascular risk assessment using QRISK3 assessment tool, and those with 10% or greater 10-year risk of developing CVD should be offered HMG-CoA reductase inhibitor (“statin”) therapy for primary prevention ^89,91^.

While statins confer survival benefit in both primary and secondary prophylaxis of cardiovascular events ^130^, their use in NAFLD is sometimes limited by concerns about hepatotoxicity. A pairwise meta-analysis of over 120,000 people in whom the presence of NAFLD was not recorded reported a small increase in liver dysfunction (OR 1.33 (1.12- 1.58)) overall, but these and other adverse effects did not outweigh the reduction in risk of major cardiovascular events ^131^. Conversely, there is evidence of benefit in the context of liver disease: in patients undergoing biopsy for suspected NASH, statin use conferred dose-dependent protection against liver-related histological endpoints, including steatohepatitis and fibrosis ^132^. A cross-sectional study of individuals with biopsy-proven NAFLD and T2DM demonstrated that statins were negatively associated with steatohepatitis and significant fibrosis in multivariate analyses ^133^. A recent meta-analysis of eight studies including patients with mixed aetiologies of cirrhosis (n=3195) concluded that statin use was associated with an improvement in portal pressure gradients and a reduced risk of variceal haemorrhage ^134^.

Therefore, we recommend patients who are at increased cardiovascular risk (T2DM and/or QRISK-3 >10%) should be offered HMG-CoA reductase inhibitor (“statin”) treatment in accordance with NICE guidelines (Recommendation 28, Table 1). Statins should not be withheld from patients with NAFLD, including patients with compensated cirrhosis, because hepatotoxicity is very rare and the benefits are likely to significantly outweigh their risks (Recommendation 29, Table 1).

##### Type 2 diabetes

Weight loss and cardiovascular risk reduction are critical components of the management of patients with both NAFLD and T2DM. There is robust evidence to demonstrate the cardiovascular benefit of specific classes of glucose lowering agents, including glucagon-like peptide-1 receptor agonists (GLP-1RA) and sodium-glucose co-transporter 2 inhibitors (SGLT2i) ^135^. In addition, both classes of agent promote weight loss and may have a beneficial impact on the liver ^136,137^. In line with published guidance ^138^, we would advocate a low threshold for the preferential use of agents that lower weight and reduce cardiovascular risk in patients with NAFLD to treat their diabetes. Accordingly, in people with NAFLD and type 2 diabetes, treatment with glucose lowering agents that promote weight loss and reduce cardiovascular risk should be considered (recommendation 30, Table 1).

#### Service considerations

##### Research and clinical trials

Patients with NAFLD should be considered for research studies and offered the opportunity to participate in clinical trials where available (Recommendation 31, Table 1). It is recognised in other disease areas that increased participation in research is associated with improved clinical outcomes for patients ^139^. NAFLD is a relatively recently described entity with substantial unmet need in terms of understanding of its natural history, diagnostic tests, and treatment to prevent disease progression. These needs can only be addressed through the engagement of people with NAFLD with research. Where research studies are available, these should be offered to appropriate patients to consider participation.

##### Multidisciplinary care for people with NAFLD

Given the strong relationship between NAFLD and the metabolic syndrome, patients with fatty liver frequently have associated metabolic and cardiovascular comorbidity and a holistic approach to their management is advised. Comprehensive management of NAFLD requires expertise in clinical hepatology for the diagnosis, staging of NAFLD and management of hepatic comorbidity. Diabetes is often present in advanced NAFLD and this, with optimisation of cardio-metabolic risk factors requires relevant expertise. Lifestyle intervention and health promotion are required to assist sustainable health improvement. International guidelines highlight the multi-disciplinary nature of interventions in NAFLD ^17,140^ and the feasibility and utility of a multi-disciplinary clinic has been demonstrated ^141^. Therefore, management of patients with advanced NAFLD in secondary care should be by multidisciplinary teams with expertise in clinical hepatology, management of diabetes and cardiovascular risk factors, lifestyle intervention and health promotion (diet and exercise/physical activity) (Recommendation 32, Table 1).

##### Triggers for re-referral to secondary care

In those discharged to primary care, recommendations should be made on triggers for re-referral back to secondary care liver services (Recommendation 33, Table 1). A baseline assessment cannot adequately exclude the future possibility of fibrosis progression and liver-related outcomes, particularly as patients might accumulate with time further risk factors and metabolic comorbidities. There is also the possibility of a false negative baseline fibrosis assessment that could lead to an inappropriate patient discharge to primary care. Therefore, there should also be systems in place for the re-evaluation of fibrosis in patients with NAFLD discharged to primary care. The method of fibrosis assessment should be based on local availability and expertise and some patients may require specific follow-up recommendations: for example, it would be inappropriate to use the FIB-4 score to follow-up a patient with non-liver related thrombocytopenia because this could lead to a false positive result. Moreover, it may be appropriate to suggest no further fibrosis assessments in patients who have significant comorbidities or frailty where management of liver disease would not alter their long-term outcomes.

Other potential triggers for re-referral might include a significant increase in serum liver enzyme values and/or laboratory indicators of advanced chronic liver disease, such as decreasing albumin, increased prothrombin time and increased bilirubin. Moreover, the development of T2DM should prompt a fibrosis reassessment because this has been associated with progression of NAFLD ^37^.

##### Provision of information about NAFLD for patients

Patients should be provided with written information about NAFLD and weight management in a format appropriate to their needs and signposted to other credible sources of information such as the NHS and the British Liver Trust (Recommendation 34, Table 1). It is important that high quality information is provided that is based on reliable and up to date evidence. This should be provided in a way that patients understand, is easy to use and navigate. Patients should be asked if they have understood the information and prompted to ask further questions. People often feel overwhelmed with information and patients report that they need it to be provided on multiple occasions and in different ways. A 2020 British Liver Trust of over 2000 liver disease patients ^142^, found that nine out of ten people tried to find out more about their condition after leaving their clinic appointment with over 90% of these looking on the internet so it is important to provide information that can be taken away and to signpost to credible information. Useful web resources include www.britishlivertrust.org.uk, the NHS website (www.nhs.uk) ^143^ and the NAFLD patient guidelines from EASL ^144^

### Conclusion

The evidence base for investigation and management of NAFLD is large and growing, but there is currently little practical guidance to support development of services and delivery of care. To address this, we have produced a series of evidence-based quality standard recommendations for the management of NAFLD, with the aim of driving improvement of the care of patients with this common condition. Currently there is no high-quality evidence for liver specific management in NAFLD, so the recommendations are likely to evolve as evidence accumulates. It is hoped that services will review their practice against the KPIs and institute service development where needed. The NAFLD SIG will aim to conduct a national audit of the management of NAFLD using the KPIs as a benchmark to promote service development.

## Figures and Tables

**Table 1:** A Summary of the NAFLD quality standard recommendations

Management of people with, or at risk of, NAFLD before the gastroenterology or liver clinic	Quality of evidence	Agreement	Responses
1. Services should have an agreed local clinical pathway for the investigation of suspected liver disease that includes an assessment for liver fibrosis using available non-invasive liver fibrosis tests	Low	100%	100% SA
2. Consider the possibility of liver fibrosis due to NAFLD in those with Type 2 diabetes (T2DM) or the metabolic syndrome	Low	96%	70% SA, 26% A, 4% N
3. Do not rely on abnormal liver blood tests to prompt consideration of liver disease. However, persistently unexplained abnormal liver blood tests should always be investigated.	Low	96%	67% SA, 29% A, 4% N
4. The finding of liver steatosis on ultrasound, or unexplained abnormal liver blood tests, should prompt risk assessment for liver fibrosis.	Low	100%	89% SA, 11% A
5. Use validated widely available non-invasive tests (e.g. FIB-4 score or NAFLD fibrosis score) with high negative predictive value to risk assess for significant liver fibrosis in the community.	Moderate	100%	85% SA, 15% A
6. Refer patients stratified as high risk for advanced fibrosis or cirrhosis to a hepatologist. For patients stratified as indeterminate risk, offer further discriminatory tests (such as transient elastography or ELF test) or refer for further evaluation.	Low	100%	67% SA, 33% A
7. Manage those at low risk of significant fibrosis in the community, with focus on lifestyle advice and cardiovascular risk reduction. Reassess fibrosis using non-invasive tests after 3 years.	Low	100%	62% SA, 38% A
8. Secondary care liver services and community services should collaborate on audit, research and education to share knowledge, strengthen links and encourage service and quality improvement and involve patients as part of this as appropriate.	Not graded	92%	SA 69%, 23% A, 8% N
**Assessment and investigations in secondary care**		**Agreement**	**Responses**
9. Patients with NAFLD should be assessed for additional causes of steatosis (e.g., drugs and alcohol) and undergo investigations for other causes of liver disease (i.e., completion of blood aetiology screen) if not already undertaken in primary care.	Low	100%	85% SA, 15% A
10. Patients with NAFLD should have a detailed alcohol history (such as AUDIT-C), illicit drug and smoking history documented.	Moderate	100%	67% SA, 33% A
11. Practitioners should document a treatment history and medicines use review. The rationalisation of medicines that may accelerate disease progression should be considered	Low	100%	65% SA, 35% A
12. An assessment of dietary habits and physical activity levels should be obtained.	Low	93%	67% SA, 26% A, 7% N
13. Patients with NAFLD should undergo sequential use of a simple non-invasive test (e.g. FIB-4) and specialist non-invasive tests (e.g. ELF, transient elastography or ARFI) to assess the severity of fibrosis	Moderate	96%	69% SA, 27% S, 4% D
14. Patients with NAFLD should be considered for a liver biopsy: A. if there is diagnostic uncertainty (other aetiologies/overlap conditions); B. to evaluate the severity of NASH and be considered for potential drug therapies (including clinical trials); or C. to determine the stage of liver fibrosis where non-invasive tests are inconclusive to aid with future management (e.g. F4 for HCC surveillance).	Moderate	92%	50% SA, 42% A, 8% N
15. Liver biopsies should be processed, stained and examined according to the RCPath guidelines and reported by pathologists who participate in the liver EQA scheme using a validated score such as the NASH CRN (NAS) or SAF score.	Low	96%	56% SA, 40% A, 4% N
16. Patients with NAFLD cirrhosis should be offered surveillance for complications of cirrhosis, including HCC and varices, in accordance with national/international recommendations. The Baveno VI exclusion criteria should be considered as a non-invasive tool to rule out the presence of varices requiring treatment.	Moderate	100%	79% SA, 21% A
17. People with NAFLD should undergo systematic assessment of cardiovascular risk factors including use of an objective risk score. (e.g. QRISK-3).	High	96%	55% SA, 41% A, 4% N
18. Patients with NAFLD should be screened annually for Type 2 Diabetes (using HbA1c), hypertension and dyslipidaemia.	Low	85%	46% SA, 39% A, 11% N, 4% SD
**Management in secondary care**		**Agreement**	**Responses**
19. People with NAFLD should be asked about smoking and, if they smoke, should be advised to stop and offered referral to smoking cessation services.	High	100%	67% SA, 33% A
20. People with NAFLD should be advised on benefits of regular exercise; a baseline assessment of physical activity should be made and individualised advice given to increase physical activity.	Moderate	92%	78% SA, 14% A, 4% N, 4% SD
21. Patients with NAFLD should have a regular reassessment of their alcohol consumption.	Low	100%	50% SA, 50% A
22. Abstinence from alcohol should be strongly recommended to patients with NAFLD and cirrhosis. Patients with pre-cirrhotic NAFLD should be advised that alcohol consumption may accelerate disease progression and so should minimise or abstain from alcohol to reduce the risk of disease progression.	Low	100%	69% SA, 31% A
23. Tailored dietary advice should be given with the aim of 5-10% body weight loss through a calorie deficit including, but not limited to, reduction of refined carbohydrates and processed foods, and increased consumption of vegetables, lean protein sources and fish. Referral to weight management services should be considered, especially if weight loss goals have not been achieved.	Low	100%	54% SA, 46% A
24. Referral for consideration of bariatric surgery should be considered in NAFLD patients with obesity who meet the eligibility criteria for bariatric surgery according to national recommendations.	Moderate	96%	50% SA, 46% A, 4% D
25. People with NAFLD who are at significantly increased risk of disease progression and potential risk of liver related complications should continue to be managed in the secondary care setting. Such patients include those with cirrhosis or significant-advanced fibrosis whose liver disease is not outweighed by comorbidities or performance status.	Low	100%	42% SA, 58% A
26. Patients with decompensated liver disease caused by NAFLD should be considered for transplant assessment.	Moderate	96%	78% SA, 18% A, 4% N
27. Patients with hypertension should be managed in accordance with NICE guidelines.	High	100%	76% SA, 24% A
28. Patients who are at increased cardiovascular risk (T2DM and/or QRISK-3 >10%) should be offered HMG-CoA reductase inhibitor (“statin”) treatment in accordance with NICE guidelines.	High	100%	76% SA, 44% A
29. Statins should not be withheld from patients with NAFLD, including patients with compensated cirrhosis, because hepatotoxicity is very rare and the benefits are likely to significantly outweigh the risk.	Moderate	100%	64% SA, 36% A
30. In people with NAFLD and type 2 diabetes, treatment with glucose lowering agents that promote weight loss and reduce cardiovascular risk should be considered.	Moderate	96%	77% SA, 19% A, 4% N
31. Patients with NAFLD should be considered for research studies and offered the opportunity to participate in clinical trials where available.	Not graded	100%	85% SA, 15% A
32. Management of patients with advanced NAFLD in secondary care should be by multidisciplinary teams with expertise in clinical hepatology, management of diabetes and cardiovascular risk factors, lifestyle intervention and health promotion (diet and exercise/physical activity)	Low	92%	58% SA, 34% A, 4% N, 4% D
33. In those discharged to primary care, recommendations should be made on triggers for re-referral back to secondary care liver services.	Low	100%	65% SA, 35% A
34. Patients should be provided with written information about NAFLD and weight management in a format appropriate to their needs and signposted to other credible sources of information such as the NHS and the British Liver Trust	Not graded	100%	65$ SA, 35% A

SA = Strongly Agree; A = Agree; N = Neutral; D = disagree; SD = strongly disagree.

**Table 2:** Auditable key performance indicators (KPIs) for the management of patients with suspected NAFLD

Quality Indicator	Minimum standard	Aspirational standard
**Management of people with, or at risk of, NAFLD before the gastroenterology or liver clinic**		
1. Services should have an agreed local clinical pathway for the investigation of suspected liver disease that includes an assessment for liver fibrosis using available non-invasive liver fibrosis tests	100%	N/A
2. Individuals referred to secondary care with suspected NAFLD should have their non-invasive fibrosis staging (e.g. FIB-4 score or NAFLD fibrosis score) documented in the referral letter.	90%	100%
**Investigations and management in secondary care**		
3. People with NAFLD should have their weight and BMI documented	90%	100%
4. People with NAFLD should have an alcohol history documented and advice given, where appropriate	90%	100%
5. People with NAFLD should have a smoking history documented and advice given, where appropriate	90%	100%
6. People with NAFLD should undergo liver fibrosis staging using available non-invasive tests or liver biopsy	90%	100%
7. Patients with NAFLD should be screened for Type 2 diabetes	90%	100%
8. People with NAFLD should be screened for Hypertension	90%	100%
9. Patients with NAFLD should have weight loss advice documented including objective goals for weight change and physical activity.	90%	100%
10. Patients who are at increased cardiovascular risk (T2DM and/or QRISK-3 >10%) should be offered statin treatment in accordance with NICE guidelines.	90%	100%
11. Patients should be provided with written information about NAFLD and weight management and/or signposted to credible information sources.	90%	100%
